# Difference in badminton‐specific endurance evaluated by a newly developed on‐court test between competitive levels: A pilot study of female players

**DOI:** 10.14814/phy2.16058

**Published:** 2024-05-20

**Authors:** Ryosuke Ando, Yoshihiro Hoshikawa, Taro Iizuka, Masashi Suita, Mai Kameda, Hirotaka Nakashima, Hiroki Ozaki

**Affiliations:** ^1^ Department of Sport Science and Research Japan Institute of Sports Sciences (JISS) Tokyo Japan; ^2^ Department of Sports Science Japan Women's College of Physical Education Tokyo Japan; ^3^ Nippon Badminton Association Tokyo Japan; ^4^ Faculty of Health and Sport Sciences University of Tsukuba Tsukuba Japan

**Keywords:** aerobic capacity, agility, blood lactate concentration, lunge step, racket sports

## Abstract

We developed a test to evaluate badminton‐specific endurance. The study included 10 female badminton players. Five participants were ranked in Japan's top 100 national rankings (ranked), whereas the others were unranked (unranked). Participants reacted quickly with badminton‐specific steps from the base center to the four sensors at each corner of a singles badminton court. On each set, they reacted eight times to randomized instructions at stage‐specific intervals (1.2, 1.0, and 0.8 s for stages 1, 2, and 3, respectively), which were performed six times with a rest of 20 s in each stage (8 movements × 6 sets × 3 stages). On a different day, participants ran on a treadmill as a comparative test. Blood lactate concentration (BLa) was measured on each test. In the badminton‐specific test, ranked participants had lower BLa (4.2 ± 1.7 mM vs. 6.3 ± 3.1 mM), with medium or large effect sizes. The average reach time to sensors was shorter in ranked participants (1.56 ± 0.03 s vs. 1.62 ± 0.07 s), with medium or large effect sizes. BLa was similar between groups, with trivial or small effect sizes in the running test. These results suggest that the newly developed test can evaluate badminton‐specific endurance.

## 1 INTRODUCTION

Badminton is one of the most physically demanding racket sports in the world and requires energy provided by both aerobic (60%–70%) and anaerobic (30%) systems (Phomsoupha & Laffaye, 2015). Recently, Fu et al. (2021) indicated that more than 90% of the energy during a singles match was provided by the aerobic system. Therefore, improving endurance may be beneficial to competitive players' badminton performance. However, Ooi et al. (2009) found no significant difference in the 20‐m multistage shuttle run between elite and sub‐elite Malaysian badminton players. We periodically assessed the blood lactate concentration (BLa) curves during treadmill running in elite and sub‐elite Japanese badminton players over two decades, which were not significantly different between competitive levels (unpublished data). These findings indicate that the competitive level of badminton could not be statistically correlated with endurance evaluated by the conventional running test.

However, two previous studies have shown significant correlations between the results of custom‐made badminton‐specific on‐court endurance tests and competitive levels. For example, Madsen et al. (2016) reported that the results of the on‐court endurance test developed by the authors correlated with players' national singles rankings in Denmark, whereas those of the Yo‐Yo intermittent recovery test (i.e., conventional running test) did not. Chin et al. (1995) measured BLa, heart rate (HR), and maximum performance (number of levels achieved) in their original on‐court endurance test with incremental loads. Their original index of three measurements correlated with the competitive level of badminton. The correlation between on‐court endurance test results and competitive level may be due to badminton‐specific steps. Badminton has the characteristic that the lunge steps occupy approximately 15%–25% of all the steps in the game (Abdullahi & Coetzee, 2017; Kuntze et al., 2010), which are asymmetrically performed with the dominant leg on the same side as the racket grip. Lam et al. (2018) showed that skilled female badminton players experienced lower peak vertical and horizontal ground reaction forces during lunge steps than their unskilled counterparts did. This suggests that skilled players can efficiently perform lunge steps in terms of energy expenditure, whereas unskilled players can impose higher energy demands. Therefore, results of badminton‐specific on‐court endurance tests were related to competitive levels in previous studies (Chin et al., 1995; Madsen et al., 2016).

Previous studies adopted continuous exercises (e.g., 3 min of badminton‐specific exercise with a rest of 45 s and a work:rest ratio of 4:1 or 30–12 s of badminton‐specific exercise with a rest of 10 s and a work:rest ratio of approximately 3:1–1:1) with all‐out efforts in their on‐court endurance tests (Abián‐Vicén et al., 2021; Chin et al., 1995; Madsen et al., 2016). However, badminton games have been characterized as submaximal load, such as 54%–80% of peak oxygen uptake and 1.9–4.3 mM for BLa (Faude et al., 2007; Fernandez‐Fernandez et al., 2013; Green et al., 2023), and intermittent exercises (Phomsoupha & Laffaye, 2015), such as 4.8–10.4 s of rally duration with rest of 8.1–26.7 s (i.e., work: rest ratio is approximately 1:2; Abdullahi & Coetzee, 2017; Gawin et al., 2015). As the exercise load and/or work:rest ratio affect energy metabolism during interval exercise (Gosselin et al., 2012; Nicolo et al., 2014), a badminton‐specific endurance test that simulates the actual game conditions should be developed.

We developed a new practical badminton‐specific endurance test that simulated the load and tempo of badminton games to evaluate badminton‐specific endurance. This study aimed to compare badminton‐specific endurance at competitive levels. We hypothesized that badminton‐specific endurance, as evaluated by the test, is superior in highly competitive players.

## 2 MATERIALS AND METHODS

### 2.1 Experimental design

Participants performed both custom‐made badminton‐specific endurance tests on a court and a step‐running test on a treadmill as a conventional test, in random order on separate occasions. Primary outcomes (BLa, HR, reach time, and ratings of perceived exertion [RPE]) were compared between ranked and unranked participants. Participants were instructed to refrain from strenuous exercise on the testing date and from resistance training 48 h before testing.

### 2.2 Participants

Ten female collegiate badminton players participated in the study. Being on the same team meant that they had similar strength training programs. Five players were champions in a Japanese intercollegiate championship in a certain year and were ranked in the top 100 in the national rankings in Japan (ranked, age: 20 ± 1 years, height: 162.4 ± 6.3 cm, body mass: 55.3 ± 3.2 kg), while other players did not participate in the championship and were unranked (unranked, age: 20 ± 1 years, height: 163.1 ± 5.8 cm, body mass: 57.9 ± 5.3 kg). Before proceeding with the experiment, the purpose of the study, its procedures, and associated risks were explained to all participants, and written informed consent was obtained. The experimental protocols were approved by the Ethics Committee of the Japan Institute of Sports Sciences (no. 2020‐040). The study was conducted in accordance with the principles of the Declaration of Helsinki.

### 2.3 Badminton‐specific endurance test

**FIGURE 1:**
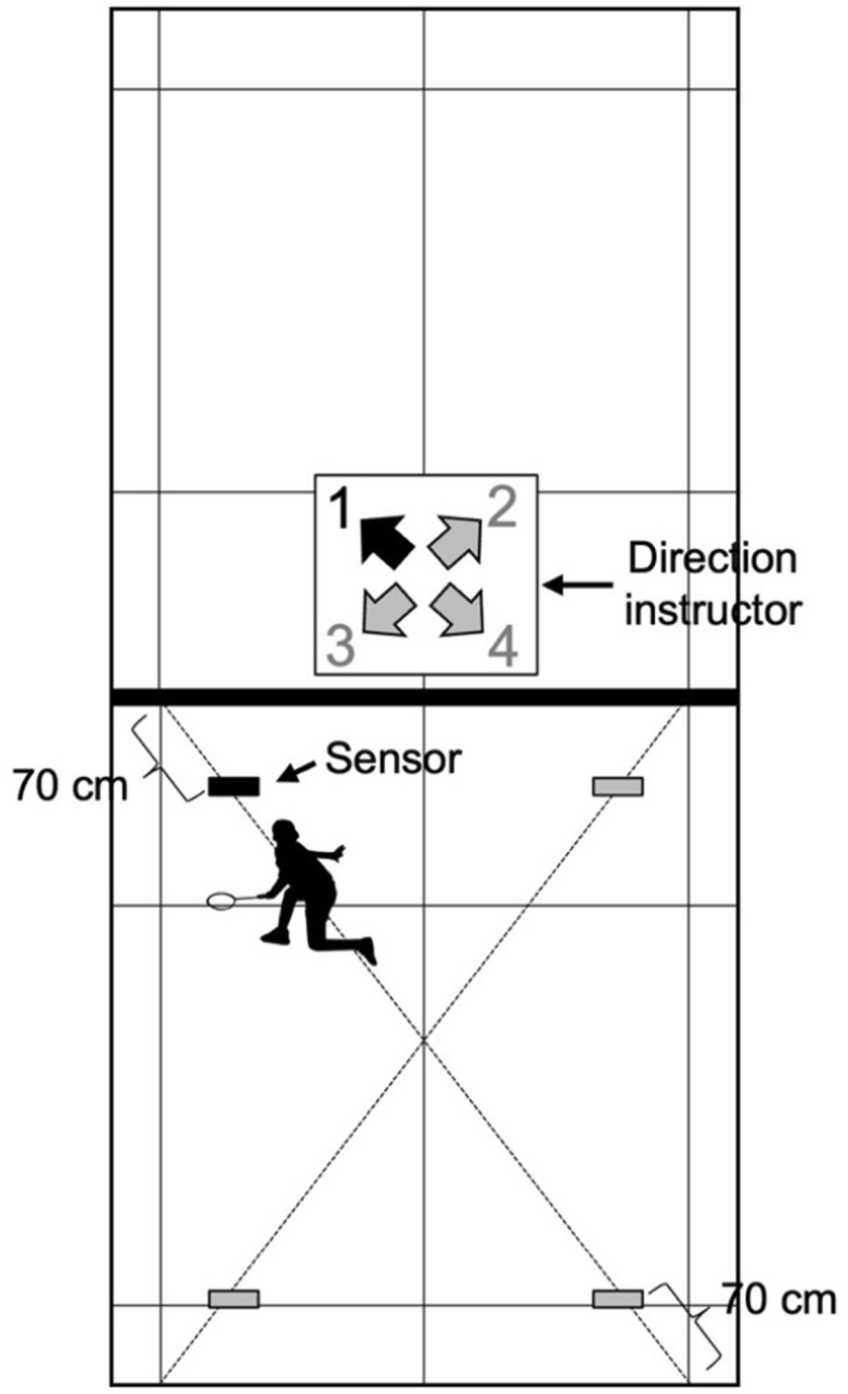
Overall view of the setup for the badminton‐specific test on the badminton court. Four sensors were located 70 cm from the diagonal end of a single court.

Four custom‐made sensors (4Assist Co. Ltd., Tokyo, Japan) were placed at the four corners (front right, front left, back right, and back left) of a singles badminton court. The details of the sensor positions are shown in Figure 1. The front and back sensors were set at the participant's knee and elbow heights, respectively. Participants started the test positioned at the base center of the court. Subsequently, participants moved toward one of the sensors, as instructed by a computer program (4Assist Co. Ltd.), with badminton‐specific movements, held their dominant hand over the sensor as fast as possible, and then returned to the base center. The base center was not defined for the participants to avoid awkward movements. The sensor to be touched was instructed on a monitor placed at the net in the middle of the court, and next direction instructions were displayed 1.5, 1.2, 1.0, or 0.8 s after holding the hand over the sensor (instruction time: details are described below). These instruction times were determined by simulating the average tempo (shots per rally time) of Singles match (0.89–1.30; Abdullahi & Coetzee, 2017; Iizuka et al., 2020; Laffaye et al., 2015). One set consisted of eight movements (2 × 4 sensors), and the instruction order of the direction was randomly generated using a computer program.

**FIGURE 2:**
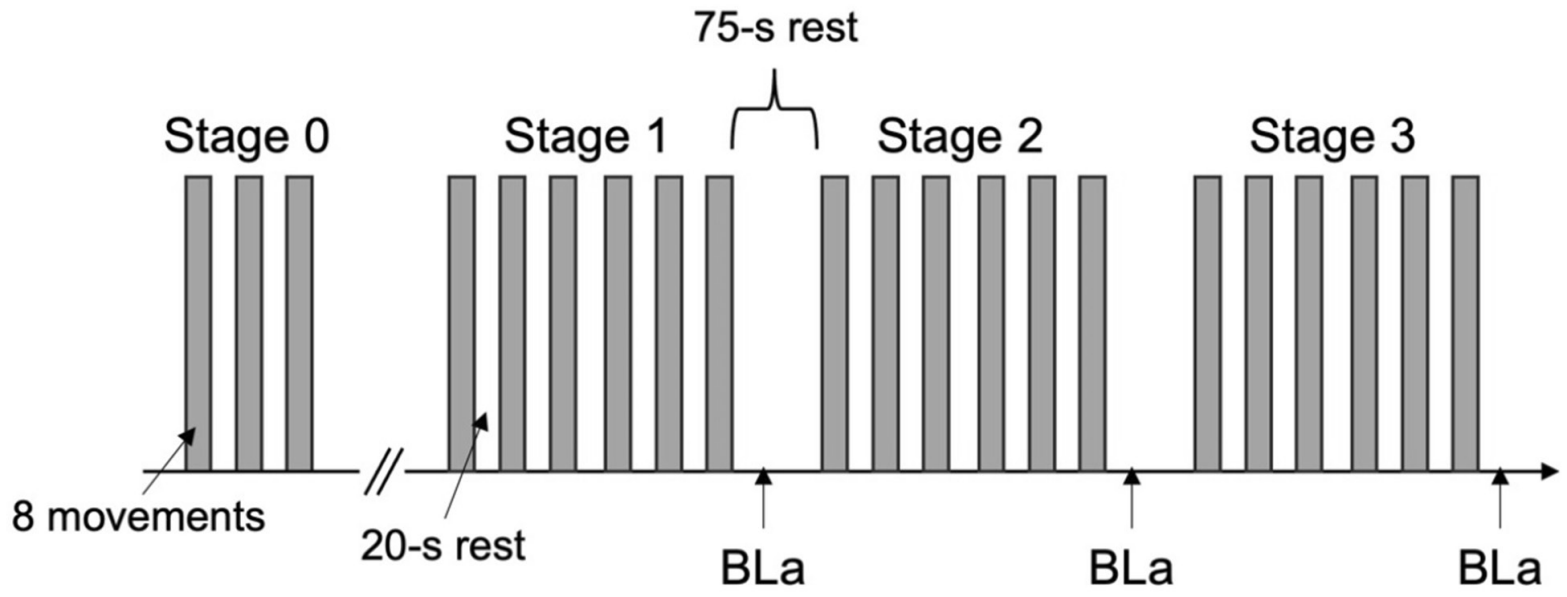
Badminton‐specific endurance test protocol.

After the self‐warm‐up, the participants performed three sets with a rest period of 20 s (stage 0). The instruction time was 1.5 s, which was sufficiently long to return to the base center and concentrate on moving as fast as possible. These three sets were performed to confirm the potential individual's movement speed (i.e., reach time, as described below). We confirmed that the HR returned to the resting value (3–5 min) after stage 0, and then started the main protocol (stages 1, 2, and 3; Figure 2).

The participants performed six sets with a rest of 20 s, which were further repeated three times (i.e., stages 1, 2, and 3) with a rest of 75 s. The rest period between sets was determined based on the average rest time in badminton match analyses (Abdullahi & Coetzee, 2017). Exercise intensity was increased by shortened instruction times, such as 1.2, 1.0, and 0.8 s for stages 1, 2, and 3, respectively. Blood samples for BLa analysis were collected from fingertips at each stage. BLa was measured 30 s after the completion of six sets on each stage using a lactate analyzer (Lactate Pro 2; Arkray, Kyoto, Japan). The RPE (Borg scale: 6–20) was assessed after each stage. The HR was recorded continuously using an HR monitor (H10, Polar, Kempele, Finland). Reach times from the display of the direction instruction to holding the hand for a sensor were averaged at each stage, which were interpreted as movement speed and were used for further analysis. As the participants could infer the last direction instruction for each set, the reach times were excluded from the average calculation. The test was completed within 20 min.

It is well known that BLa is dependent on exercise intensity. Standardizing exercise intensities was insufficient due to variations in individual reach times. Therefore, we calculated the deviation score (*T*‐score) for BLa and reach time at each stage in our participants, meaning six *T*‐scores were calculated for each participant. These were averaged for each participant (average *T*‐score). Because lower values indicated superiority for both BLa and reach time, the *T*‐score was calculated such that lower values of BLa and reach time indicated higher *T*‐scores.

Test–retest reliability for BLa and reach time in the badminton‐specific endurance test was confirmed in eight female players ranked in the top 100 in the national rankings in Japan. They performed only stage 2 (instruction time is 1.0 s) twice on different occasions, 2 months apart. Between‐day coefficients of variation for BLa and reach time were 11.9 ± 8.3% and 1.5 ± 0.8%, respectively.

### 2.4 Running test

The conventional running test was performed on a computer‐controlled treadmill (BM‐1200; S & ME Co. Ltd., Tokyo, Japan). After self‐warm‐ups, each participant performed the step exercise test, which consisted of three levels of steps (10.8, 12.6, and 14.4 km·h^−1^ of running velocity) with no slope angle (0%). The reasons for selecting these running velocities were that BLa was approximately 2–5 mM in elite female badminton players (unpublished observation), and these BLa values were similar to badminton games (1.9–4.3 mM; Faude et al., 2007; Fernandez‐Fernandez et al., 2013; Green et al., 2023). The duration of each step and the rest time between steps were 3 and 1 min, respectively. Blood samples were collected from the fingertips after each step. The samples were analyzed using a lactate analyzer (Lactate Pro 2). The RPE (Borg scale: 6–20) was assessed after each step. HR monitor (H10, Polar) continuously recorded HR.

### 2.5 Statistical analyses

Statistical analyses were performed using IBM SPSS Statistics software (version 29.0; IBM, Armonk, NY, USA). Normality was tested using the Shapiro–Wilk test, which did not indicate normality for the data of some parameters. Furthermore, the sample size was small. Therefore, we used a nonparametric test in this study. BLa, average HR, reach time, RPE, and average *T*‐score were compared between ranked and unranked players using the Mann–Whitney *U*‐test, and the effect size (*r*) was calculated. The value of *r* was interpreted as *r* < 0.10 for the trivial effect, 0.10 ≤ *r* < 0.30 for the small effect, 0.30 ≤ *r* < 0.50 for the medium effect, and 0.50 ≤ *r* for the large effect (Cohen, 1988). All data are presented as a mean ± SD. The level of significance was set at *p* < 0.05 for all analyses.

## 3 RESULTS

**FIGURE 3:**
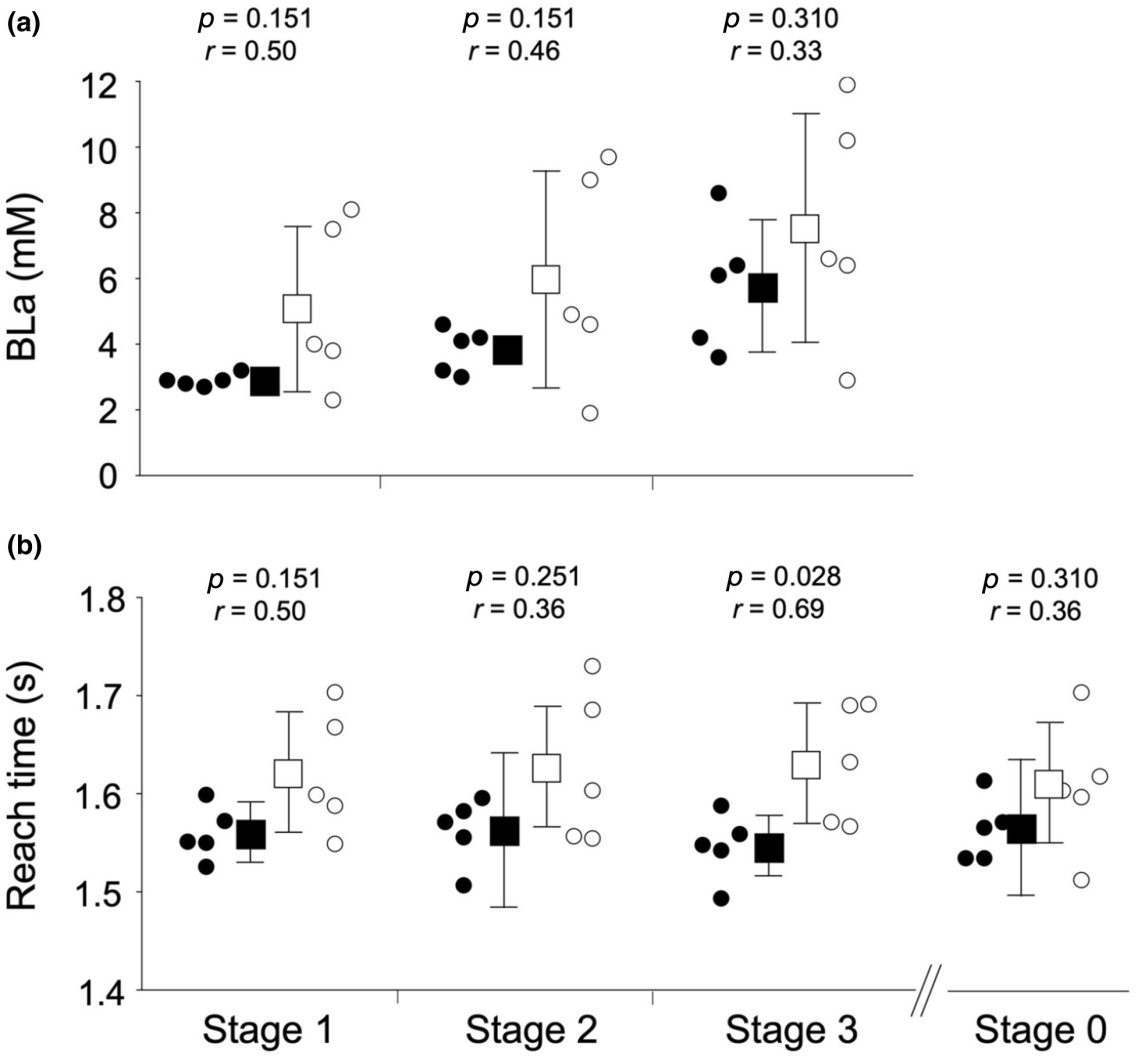
Blood lactate concentration (BLa) (a) and reach time (b) for ranked and unranked players in the badminton‐specific endurance test. The solid circle indicates individual plots of ranked players, the solid square indicates the average values of ranked players with SDs, the open circle indicates individual plots of unranked players, and the open square indicates the average values of unranked players with SDs.

**FIGURE 4:**
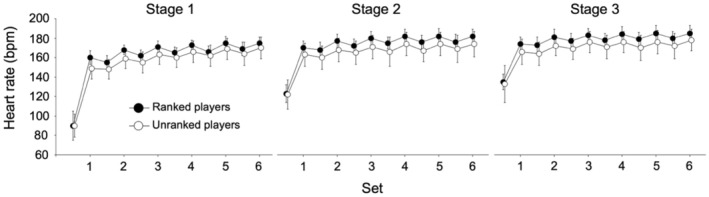
Changes in heart rate during the badminton‐specific endurance test.

**FIGURE 5:**
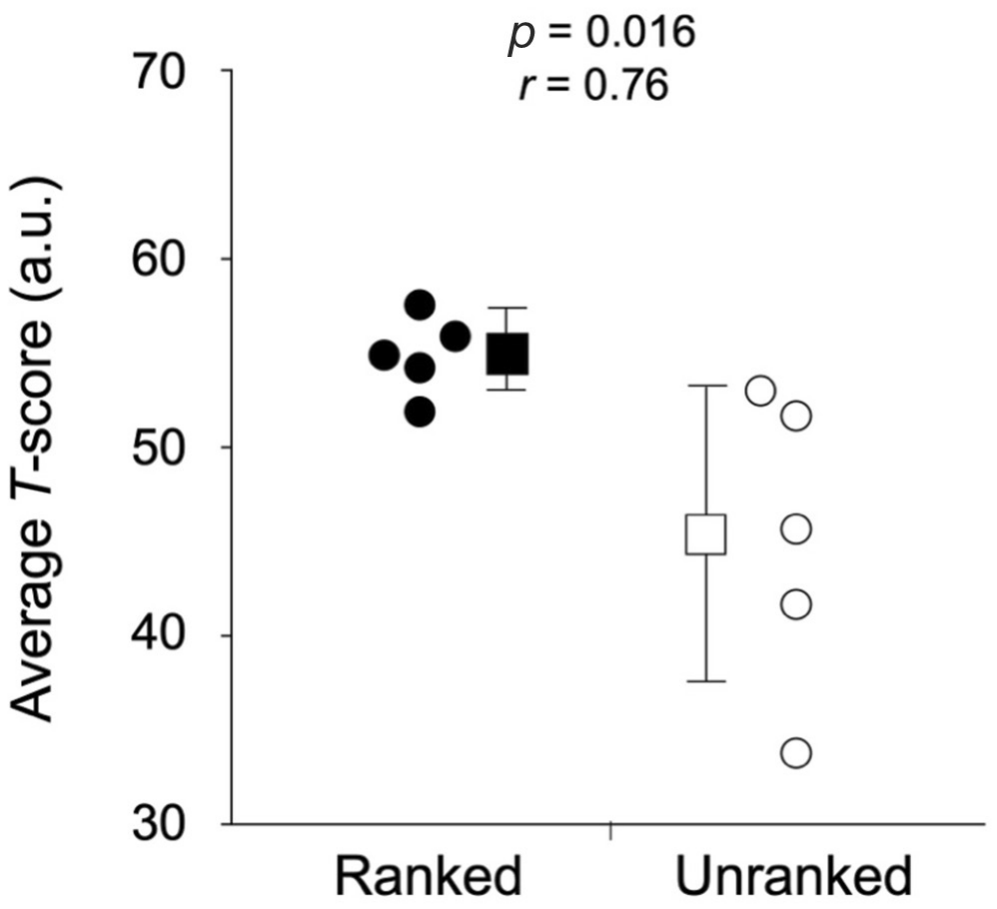
Average *T*‐score for ranked and unranked players in the badminton‐specific endurance test. The solid circle indicates individual plots of ranked players, the solid square indicates the average value of ranked players with SD, the open circle represents individual plots of unranked players, and the open square represents the average value of unranked players with SD.

The BLa used in the badminton‐specific endurance test is shown in Figure 3a. BLa was not significantly different between competitive levels, with medium or large effect sizes at all stages. Reach time was significantly shorter in ranked players than in unranked players at stage 3 with a large effect size, while it was not significantly different between them at stages 0, 1, and 2 with medium or large effect sizes (Figure 3b). Changes in HR during badminton‐specific endurance tests are shown in Figure 4. The average HR and RPE values during the badminton‐specific endurance test are shown in Table 1. There were no significant differences in the average HR between ranked and unranked players, with trivial or small effect sizes at all stages. The RPE did not differ significantly between the ranked and unranked players, with small or medium effect sizes at all stages. The average *T*‐score was significantly greater in ranked players than in unranked players with a large effect size (Figure 5).

**TABLE 1:** Averaged HR and RPE during the badminton‐specific endurance test and the running test.

	Badminton‐specific endurance test				Running test			
	Ranked	Unranked	*p* value	Effect size (*r*)	Ranked	Unranked	*p* value	Effect size (*r*)
Averaged HR (bpm)								
Stage 1	176 ± 6	172 ± 11	0.602	0.17	148 ± 6	146 ± 12	0.916	0.03
Stage 2	182 ± 7	178 ± 14	0.834	0.07	166 ± 6	163 ± 10	0.754	0.10
Stage 3	185 ± 8	182 ± 12	0.674	0.13	178 ± 6	177 ± 11	0.465	0.23
RPE (a.u.)								
Stage 1	14 ± 2	15 ± 2	0.395	0.27	12 ± 1	12 ± 1	0.339	0.30
Stage 2	16 ± 1	17 ± 2	0.242	0.37	14 ± 1	14 ± 1	0.661	0.14
Stage 3	18 ± 2	19 ± 2	0.331	0.31	16 ± 1	16 ± 1	0.827	0.07

*Note*: Values are expressed as a mean ± SD.

Abbreviations: HR, heart rate; RPE, rating of perceived exertion.

**FIGURE 6:**
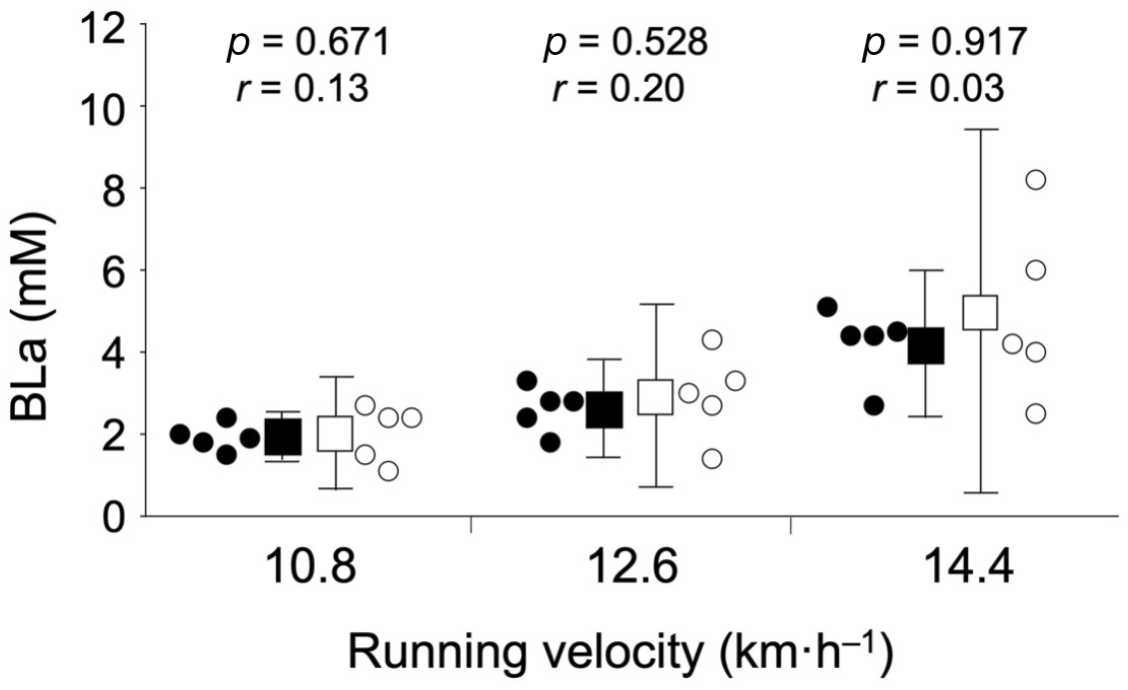
Blood lactate concentration (BLa) for ranked and unranked players in the running test. The solid circle indicates individual plots of ranked players, the solid square indicates the average values of ranked players with standard deviations, the open circle represents individual plots of unranked players, and the open square represents the average values of unranked players with SDs.

The BLa used in the running test is shown in Figure 6. The BLa was not significantly different between competitive levels with trivial or small effect sizes at any step. The average HR and RPE values during the running test are listed in Table 1. No significant differences in the average HR were observed between the ranked and unranked players, with trivial or small effect sizes at all steps. The RPE did not differ significantly between the ranked and unranked players, with trivial, small, or medium effect sizes.

## 4 DISCUSSION

In this study, we developed a new badminton‐specific endurance test and compared badminton‐specific endurance evaluated by the test between competitive levels. This study's main findings were as follows: (1) BLa and reach time tended to be superior in ranked players with medium or large effect sizes in the badminton‐specific test, (2) the average *T*‐score for BLa and reach time was significantly greater in ranked players with large effect size in the badminton‐specific test, and (3) BLa did not differ significantly between competitive levels with trivial or small effect sizes in the running test (i.e., the conventional test). These results indicate that the badminton‐specific endurance test, compared to the conventional endurance test, can evaluate badminton‐specific endurance.

The reach time for the four corners was significantly shorter in ranked players than in unranked players with a large effect size at stage 3, whereas it was not significantly different between them with medium or large effect sizes at stages 0, 1, and 2. These results suggest that ranked players can move quickly compared to unranked players on court, especially at quick rally tempos, which supports previous findings (Chin et al., 1995; Madsen et al., 2015). For example, Madsen et al. (2015) measured the time taken to finish for 20 randomized badminton‐specific actions in court. They showed that elite players were significantly faster than non‐elite and non‐badminton players, although the 30‐m sprint performance was similar among the three groups. Furthermore, Madsen et al. (2016) suggested that elite badminton players can maintain high‐intensity intermittent actions when the movement speed is gradually increased. Overall, our study and previous findings suggest that highly competitive players can move faster and repeat with a quick rally tempo.

The reach times tended to be shorter for the ranked players at all stages of the badminton‐specific endurance test. These results suggest that exercise intensity is higher in ranked players than in unranked players. This may partly explain why the difference in BLa between competitive levels did not reach statistical significance despite medium or large effect sizes. To overcome this limitation, the *T*‐score was calculated. The average *T*‐score was significantly higher in ranked players than in unranked players, indicating that ranked players moved faster with a lower BLa. In contrast, in the running test, BLa was not significantly different between ranked and unranked players, with trivial or small effect sizes at a given velocity (i.e., identical exercise intensity). These findings support the results of a previous study that showed that badminton‐specific endurance was superior at higher performance levels, whereas such a difference was not observed for the Yo‐Yo intermittent recovery test (i.e., conventional running test; Madsen et al., 2016). Therefore, ranked participants may have greater badminton‐specific endurance.

Two possible explanations account for the enhanced badminton‐specific endurance in ranked players. First, the muscle endurance capacity contributing to badminton‐specific steps may be greater in ranked players. In badminton, the lunge steps occupy approximately 15%–25% (Abdullahi & Coetzee, 2017; Kuntze et al., 2010) and are asymmetrically performed with the dominant leg on the same side as the racket grip. This characteristic could explain the greater cross‐sectional area of the gluteus maximus muscle on the preferred leg than on the non‐preferred leg in badminton players (Muramatsu et al., 2010). These findings suggest a greater contribution of the gluteus maximus muscle (i.e., hip extension) to lunge steps. Overall, the muscle endurance capacity of the gluteus maximus may be greater in ranked players than in unranked players. Second, ranked players can efficiently perform badminton‐specific steps, especially lunge steps. Previous research has shown that skilled female badminton players, compared to unskilled female badminton players, experienced lower peak vertical and horizontal ground reaction forces during lunge steps (Lam et al., 2018). These two possible reasons could be related to the greater badminton‐specific endurance of the ranked players in this study.

In the running test, BLa was not significantly different between ranked and unranked players, with trivial or small effect sizes. This result supports a previous finding showing similar endurance between elite and sub‐elite badminton players in a conventional running test (Ooi et al., 2009). Withers et al. (1981) showed that the anaerobic threshold was greater in cyclists than in runners on a bicycle ergometer and in runners than in cyclists on the treadmill. This finding indicates that aerobic capacity depends on the specificity of the competition's movement patterns. As discussed above, many asymmetrical lunge steps are involved in a badminton game. Therefore, the results of the conventional running test could not be related to the competitive levels in badminton players.

The study has three potential limitations. The rest ratio of the badminton‐specific endurance test developed (approximately 1:1) was still different from that of badminton games (approximately 1:2; Abdullahi & Coetzee, 2017; Gawin et al., 2015; Iizuka et al., 2020; Laffaye et al., 2015). Furthermore, because participants did not perform a hitting action (only holding the dominant hand over the sensor), the BLa associated with the hitting action was not considered. Therefore, the results may not completely reflect BLa during actual badminton games. However, badminton players appear to rest for less than double the rally time when playing longer rallies (e.g., 20 s; unpublished observation), meaning that the work:rest ratio of the badminton‐specific endurance test in this study could be similar to that of badminton games. Indeed, BLa was 3.0–4.6 mM in ranked players at stage 2 (similar tempo with Singles match) of the badminton‐specific endurance test, while that was 1.9–4.3 mM in badminton games (Faude et al., 2007; Fernandez‐Fernandez et al., 2013; Green et al., 2023). Therefore, our test seems to reflect endurance in badminton games well, even though it is slightly different in the work:rest ratio and lack of hitting action. Finally, test–retest reliability was assessed using data collected 2 months apart. Given the long interval between tests, the fitness levels of the participants could change. Unfortunately, we did not confirm their fitness level simultaneously. Therefore, caution is warranted when interpreting the reliability of the test–retest data, as the effect of physiological changes could be included in the between‐day coefficients of variation for BLa and reach time presented in the study.

Our results would be helpful in monitoring the condition of badminton players, especially elite players, because they spend a lot of time on skill/strength training, traveling, and participating in international tournament activities. Therefore, it would be difficult to find time for all‐out tests developed in previous studies (Abián‐Vicén et al., 2021; Chin et al., 1995; Madsen et al., 2016). This study showed that BLa increased to 3.0–4.6 mM in ranked players at stage 2 (similar tempo with Singles match) of the badminton‐specific endurance test, which is similar to BLa in badminton matches (Faude et al., 2007; Fernandez‐Fernandez et al., 2013; Green et al., 2023). Therefore, badminton players do not need to perform an all‐out test and need less than 10 min to determine their badminton‐specific endurance by performing only stage 2 of our protocol. Unfortunately, the participants were not at the international level, and further studies are warranted.

In conclusion, this study developed the badminton‐specific endurance test and compared the badminton‐specific endurance between competitive levels. In the badminton‐specific endurance test, BLa and reach time tended to be superior in highly competitive badminton players, and the average *T*‐scores for BLa and reach time were significantly higher in ranked players than those in unranked players. The BLa was similar between competitive levels during treadmill running. These results suggest that the newly developed badminton‐specific endurance test can evaluate badminton‐specific endurance.

## FUNDING INFORMATION

No funding information provided.

## CONFLICT OF INTEREST STATEMENT

The authors have no conflicts of interest to report.

## ETHICS STATEMENT

Before proceeding with the experiment, the purpose of the study, its procedures, and associated risks were explained to all participants, and written informed consent was obtained. The experimental protocols were approved by the Ethics Committee of the Japan Institute of Sports Sciences (no. 2020‐040).

## Data Availability

Data are available from the authors upon reasonable request.
